# EU MECI: A Network-Structured Indicator for a Union of Equality

**DOI:** 10.1007/s11205-023-03079-9

**Published:** 2023-02-09

**Authors:** Athanasios Lapatinas, Marina-Selini Katsaiti

**Affiliations:** 1 European Commission, Joint Research Centre (JRC), Ispra, Italy; 2 Department of Economics, University of Ioannina, Ioannina, Greece; 3 Department of Regional and Economic Development, Agricultural University of Athens, Amfissa, Greece; 4 Department of Innovation in Government and Society, College of Business and Economics, United Arab Emirates University, Al Ain, UAE; 5 Hellenic Open University, Patras, Greece

**Keywords:** Economic complexity, Equalities, Inequalities, EU, C430

## Abstract

How are the Member States performing in their challenge toward a fairer and more equal Europe? Based on the data measured by the EU Multidimensional Inequality Monitoring Framework (EU MIMF), we introduce the Multidimensional Equality Complexity Index, EU MECI, derived by structuring the EU MIMF data as a bipartite network of countries and indicators. EU MECI is defined upon the economic complexity methodology, exploiting the network’s centrality metrics to calculate aggregate scores of the capacity of Member States to ‘*build a Union of equality*’.

## Introduction

I will not rest when it comes to building a Union of equality.

         — President von der Leyen, The State of the European Union 2020

Building a Union of equality requires extensive knowledge of prevailing inequalities across multiple life domains, from material living conditions to health and from environmental conditions to political participation, social life, knowledge and education. Inequalities pose a major threat to social cohesion and the COVID-19 pandemic has inflated these by attacking the global society at its core (UN, 2020). The global health crisis calls for immediate political action to avoid a further deepening of poverty and inequalities, and in turn, political action calls for information, evidence and policy research.

Although inequality has been the focus of a vast amount of research aiming to identify policy recommendations and solutions, the consensus regarding goals, priorities and methods is often limited (Alberti et al., 2021). Different measures and methodological approaches serve as sources of possible inconsistencies that often lead to incomparable, mixed or even strikingly different results (OECD, 2017). Inequality has been measured through the use of vertical inequality metrics (Atkinson & Brandolini, 2010; Niño-Zarazúa et al., 2017; Ravallion et al., 2004), horizontal inequality measures (OECD, 2018), the equality of opportunity approach (Ferreira & Peragine, 2016), the capability approach (Sen, 1985, 1999; Nussbaum, 2001), the social mobility approach (OECD, 2017) and the discriminatory norms, attitudes and practices approach (Williamson, 2000). Despite the valuable and valid conclusions that the received literature extracts, it becomes evident that such a complex and multifaceted issue, requires a more comprehensive methodological approach, capable of synthesizing inter-related and complementary elements captured through the different approaches.

Following a request from the European Parliament, the European Commission (EC)’s Joint Research Centre (JRC) has undertaken a pilot research project to develop a Multidimensional Inequality Monitoring Framework for the EU (EU MIMF) that provides a novel and comprehensive dashboard of 346 indicators for the 28 Member States.^1^

The JRC researchers refrain from computing a composite inequality index leading to a single EU inequality ranking, due to the correlation structure of the indicators used. They offer instead an analysis based on the set of 346 indicators, recognizing though, the importance of a country-ranking methodology and providing a way to compare countries’ performances based on a double-sorting algorithm (Copeland method (Copeland, 1951)). Hence, the EU MIMF captures information from a great spectrum of dimensions with regard to inequality, arguably more informative than traditional indexes, such as the IHDI, the Gini, the OECD inequality framework, the Measurement Framework for Equality developed by the Equality and Human Rights Commission (EHRC), the Multidimensional Inequality Framework (MIF) by the London School of Economics (LSE)’s Centre for Analysis and Social Exclusion (CASE), the Sustainable Society Index by the TH Köln, among others (Alberti et al., 2021).

The EU MIMF methodology produces a ranking, able to identify “winners” and “losers” across the inequality indicators, based on pairwise comparisons between the Member States. Furthermore, the application of equal weights across Member States and indicators, results in a ranking that fails to account for the complex interconnections of the countries-indicators system.

Sciarra et al., (2020) introduce a novel mathematical approach, the *GENeralized Economic comPlexitY (GENEPY)*, based on linear-algebra tools within a bipartite-networks framework to measure economic complexity, reconciling the established methodologies of Economic Complexity Index (Hidalgo & Hausmann, 2009) and Fitness (Tacchella et al., 2012; Sciarra et al., 2021) apply this methodology to assess the Sustainable Development Goals (SDGs). The advantage of the recommended methodology lies in considering all available information on network dynamics, in both trade and SDG data, while exploiting the non-linearities inherent in such systems.

In this paper, we apply the *GENEPY* methodological approach on the inequality indicators used in the EU MIMF. Through the use of this methodology, we devise and suggest the EU Multidimensional Equality Complexity Index, EU MECI, that quantifies the overall structure of the hidden bipartite network of countries and equality indicators, beyond one-to-one comparisons, to emphasize and disentangle the intrinsic complexity of this system. To the best of our knowledge, this is the first attempt to introduce a new index, that ranks the EU Member States using network science and data-driven weights, to unveil the policy areas in which countries struggle to make progress in their efforts to tackle inequalities.

Our ranking of indicators and the EU MECI allow for a geographical prioritization of the corresponding policy interventions, pointing towards the directions in which EU and Member State strategies should align. The high correlation structure between the EU MECI, on one hand, and per capita GDP, HDI and Life Satisfaction, on the other, enforces the reliability of our proposed tool. The emerging disparities between the EU MECI and the aforementioned traditional indicators, raises questions regarding the comprehensiveness of the latter, mainly due to the data-driven weights revealed through our method, which highlight the indicators in which countries trail behind and/or struggle to improve.

The identification of obstacles to the EC president’s call for action towards a more equitable, fair and inclusive EU is the cornerstone of the 2019–2024 priority of ‘*An economy that works for people*’ for ‘*a deeper and fairer economic and monetary union*’.^2^

## The Complex Network of Member States and Equality Measures

### Data

Information on equality measures comes from the EU MIMF, which is a comprehensive dashboard of inequality-related indicators covering different dimensions of well-being and conceptual approaches for measuring inequality.^3^ The 346 indicators of the EU MIMF are calculated for the most recent year available at the time of the preparation of the framework (2021) and cover the (then) 28 EU Member States. The EU MIMF spans 10 key life dimensions: (1) Knowledge and skills, (2) Health, (3) Material living conditions, (4) Natural and environmental conditions, (5) Working life, (6) Cultural life and recreation, (7) Political participation and voice, (8) Social and family life, (9) Bodily integrity and safety, and (10) Overall life experience. Each domain covers indicators from up to five different approaches for measuring inequalities: horizontal inequality metrics, vertical inequality metrics, inequality of opportunity metrics, intergenerational mobility metrics, and metrics related to the presence of discriminatory norms, attitudes and practices (a detailed discussion of each of the different methodological approaches applied in the EU MIMF can be found in Chapter 1 of the report (Alberti et al., 2021). The full list of EU MIMF indicators is shown in the Annex of the report).

The aim of the EU MIMF project is to ‘*provide independent scientific advice on the measurement, monitoring and analysis of a wide range of different aspects of inequality, from unacceptable disparities in life outcomes to inequalities of opportunity and obstacles to social mobility*’ ((Alberti et al., 2021), p. 8). In practical terms, the EU MIMF addresses the different aspects of inequality at the EU level by bringing together relevant indicators from different measurement approaches, constructed upon ten key life dimensions.

For the analysis, all 346 listed indicators have been transformed and normalised according to a maximum (100 indicating the best performance in terms of equality) and a minimum value (0 indicating the poorest performance) to ensure comparability and enable the aggregation of measurements. Then, indicators with missing values are dropped. The final list of 177 indicators considered in the computation of the EU MECI is shown in Table 4 of the  Appendix A.

Figure 8 in the Appendix A. shows the distribution of indicators per measuring approach and key life dimensions in comparison to the initial EU MIMF set. Careful observation of the different colouring, blue and orange, representing the EU MECI and EU MIMF sets respectively, reveals that the reduction of indicators in the EU MECI is proportionally distributed across dimensions and measuring approaches, with no substantial biases. In addition, we argue that our approach, being indicator and not dimension driven, leaves the informative content of the EU MECI at dimensions’ level, qualitatively intact. The main implication of the reduced set of indicators considered in the computation of the EU MECI, as compared to the EU MIMF, is the inability of deriving valuable conclusions in the remaining missing inequality areas. Last, we note that our set of 177 indicators includes 15 out of the 16 social and labour market inequality indicators selected by the JRC researchers in their country ranking (Figure 3.2 of the EU MIMF report (Alberti et al., 2021)) as having the stronger conceptual link to the variables included in the Social Scoreboard for the European Pillar of Social Rights.^4^

### The Bipartite Network of Member States and Equality Measures

A central role in our analysis is played by the bipartite network of Member States and equality indicators. Several examples of bipartite or bi-modal networks appear in the scientific literature (Allesina & Tang, 2012; Bascompte et al., 2003; Sbardella et al., 2017). In this analysis, we use the data for the 177 indicators described above to generate a $28\times 177$ Member States–indicators matrix $\textbf{M}$, where the matrix element $M_{ci}$ represents country *c*’s achievement in equality indicator *i* (a value ranging from 0 to 100, indicating worst and best performance, respectively).

**Fig. 1:**
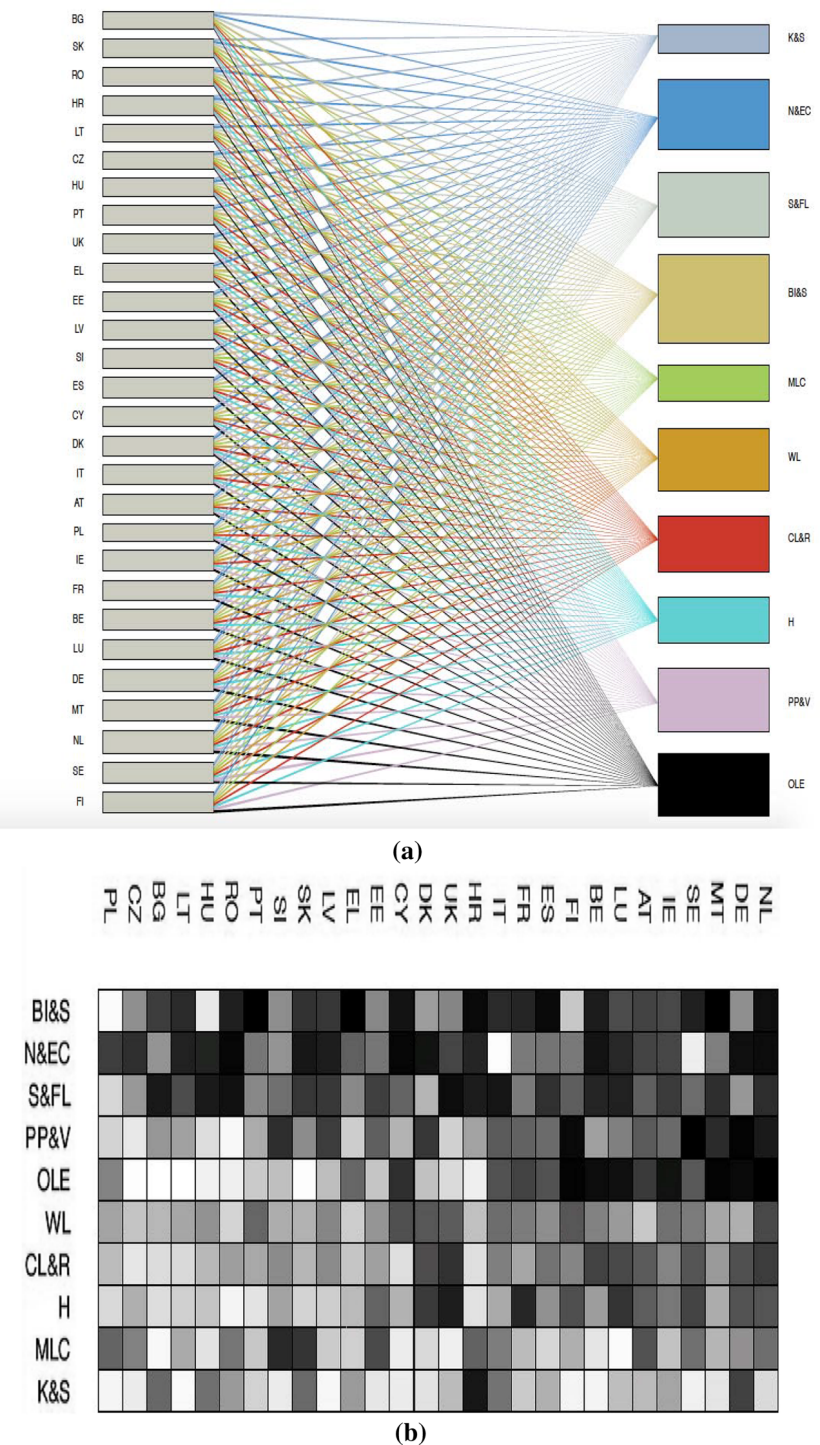
(**a**) The bipartite network of countries and equality dimensions. The 28 EU Member States are listed on the left. The 10 equality dimensions are reported on the right: $K \& S$: ‘*Knowledge and skills*’; $N \& EC$: ‘*Natural and environmental conditions*’; $S \& FL$: ‘*Social and family life*’; $BI \& S$: ‘*Bodily integrity and safety*’; *MLC*: ‘*Material living conditions*’; *WL*: ‘*Working life*’; $CL \& R$: ‘*Cultural life and recreation*’; *H*: ‘*Health*’; $PP \& V$: ‘*Political participation and voice*’; *OLE*: ‘*Overall life experience*’. The node sizes and link densities are proportional to the level of performance, i.e. a larger size indicates a higher overall (on average) performance. (**b**) The adjacency matrix using the equality dimensions. The 28 EU Member States are listed at the top. The 10 equality dimensions are reported on the left: $BI \& S$: ‘*Bodily integrity and safety*’; $N \& EC$: ‘*Natural and environmental conditions*’; $S \& FL$: ‘*Social and family life*’; $PP \& V$: ‘*Political participation and voice*’; *OLE*: ‘*Overall life experience*’; *WL*: ‘*Working life*’; $CL \& R$: ‘*Cultural life and recreation*’; *H*: ‘*Health*’; *MLC*: ‘*Material living conditions*’; $K \& S$: ‘*Knowledge and skills*’. Dark grey indicates higher aggregated values (averages) at the above 10 equality dimensions, i.e. a higher overall performance at the 10 equality dimensions

**Fig. 2:**
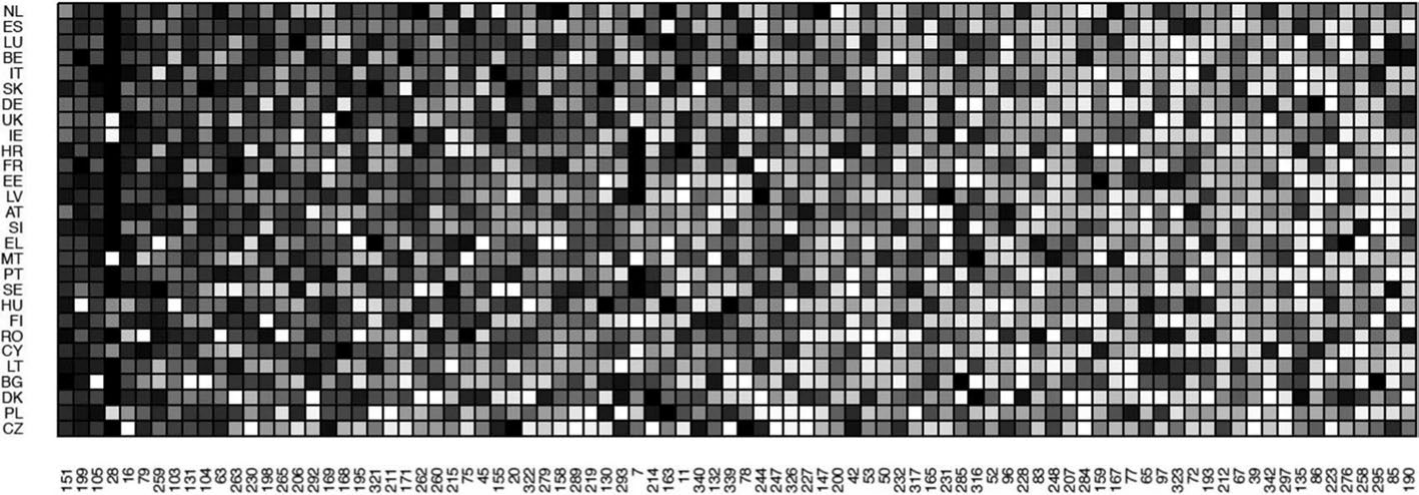
The adjacency matrix using the equality indicators. The 28 EU Member States are listed on the left. The equality indicator codes as given in the EU MIMF dataset are reported at the bottom. For ease of visualization, only the indicators with the higher 50% of average values (across countries) are shown.

The matrix $\textbf{M}$ allows for the construction of an undirected network of Member States and equality goals by linking each of the Member States to the equality indicators. Figure 1a shows the bipartite network of countries and equality measures aggregated (averages) at the 10 key life dimensions discussed above. We connect the equality indicators with each country via the performance values $M_{ci}$, according to the EU MIMF data: the denser the links, the better the (average) performance of the Member States in a given equality indicator. Figure 1b shows the respective adjacency matrix: darker squares indicate higher average values, i.e. a higher overall performance. The main equality dimensions showing high performance, on average, are ‘*Bodily integrity and safety*’, ‘*Natural and environmental conditions*’ and ‘*Social and family life*’. The dimension for which countries appear to experience most difficulty in improving their position (on average) is ‘*Knowledge and skills*’

Figure 2 presents the adjacency matrix using the equality indicators. For ease of visualisation, we only consider the indicators with average values above the median. The adjacency matrix depicts the existence of a group of Member States that performs very well in many equality indicators (the countries listed at the top of the matrix) and other Member States that perform well only in a small set of equality goals (at the bottom of the matrix). The economic complexity methodology that we discuss below assigns a higher index score to the former. With respect to the indicators, the matrix shows that code 151, ‘*Relative gap in average hours of of full-time employment worked per week (female over male)*’ in the ‘*Working life*’ dimension, followed by code 199, ‘*Odds ratio of practising artistic activities as a hobby (young adult over middle-aged adult, adjusted)*’ in the ‘*Cultural life and recreation*’ dimension and code 105, ‘*Odds ratio of household not being able to afford an unexpected expense (elderly over middle-aged adult, adjusted)*’ in the ‘*Material living conditions*’ dimension are relatively ‘easy to accomplish’ equality goals at the EU level. On the other hand, equality measures such as the ‘*Odds ratio of people rating their satisfaction with leisure time as high (female over male, adjusted)*’ (code 190) in the ‘*Cultural life and recreation*’ dimension and the ‘*Odds ratio of feeling depressed (senior over middle-aged adult, adjusted)*’ in the ‘*Health*’ dimension, seem to require more effort and better strategies from the policy side.

## The Economic Complexity Methodology

To rank the equality performance of Member States based on their equality indicator scores, we rely on complex network science and, more specifically, on the *GENEPY* methodology described in Sciarra et al. (2020) and Sciarra et al. (2021), which reconciles the commonly used methodologies to measure economic complexity, i.e. the *method of reflections* developed by Hidalgo and Hausmann (2009) and, the *fitness and complexity* algorithm developed by Tacchella et al. (2012). Economic complexity methodologies quantify the international trade network, using dimensionality reduction techniques on data capturing economic activities, without imposing the factors of production, but rather through discovering the combinations of such factors, that best explain the geography of exported goods directly from the data (Hidalgo, 2021).

Sciarra et al. (2021) take the method of Sciarra et al. (2020) one step further, by applying it on the Sustainable Development Goals (SDGs). In this case, the bipartite system consists of countries and Goals instead of countries and exports, while the achievements are eventually ranked –by Goal and by country– after allowing for complex interplays between the two. According to the authors, the *GENEPY* framework represents an appropriate methodology to assess country achievements in SDGs by (a) accounting for the relative performance of countries and the synergies and trade-offs across Goals, and (b) providing useful insights of the policy actions required to be undertaken by the countries to achieve sustainable development. It does so by assigning higher *GENEPY* scores to the few countries that have relatively more capabilities in implementing policy interventions towards achieving the Goals within the Agenda 2030.

In the same vein, we argue that the *GENEPY* methodology can be directly applicable to equality indicators. The system of 28 countries and 346 indicators within the EU MIMF is complex and characterised by high heterogeneity and disordered interactions, and any attempt to assess multidimensional equalities is not compatible with the absence of a complex networks approach (Sciarra et al., 2021; Le Blanc, 2015; Ladyman et al., 2013). In particular, equality indicators are linked through the country specific knowledge embedded in the political and economic institutions (Acemoglu et al., 2005), culture and history (Tabellini, 2008), while countries’ achievements across indicators exhibit significant heterogeneity. Thus, the double-sorting algorithm (Copeland method (Copeland, 1951)) adopted in the EU MIMF, based on one-to-one comparisons between the Member States, is unable to capture the overall structure of the bipartite network, and only considers the local information of node connections (Sciarra et al., 2021; Bonacich, 1987). In addition, even in the case of an application of the Copeland method with non-neutral weights, the *GENEPY* framework, is still arguably more comprehensive, since the weights are not imposed arbitrarily but rather are data driven.

To consolidate the multidimensional information available for the Member States into a single composite equality indicator, we devise and introduce the EU MECI, defined upon the *GENEPY* methodology, exploiting the network’s centrality metrics, to introduce an innovative way to measure and address inequalities. The advantage of this approach is that it accounts for (a) the complex interconnections between the equality indicators and (b) the high heterogeneity of Member States performance across the equality goals.

The *GENEPY* framework is set in a linear algebra framework and embeds the interplay between Member States and equality indicators (Sciarra et al., 2021, 2020) by defining the following system of $S_c$ and $Y_i$ centrality properties for countries and equality indicators, respectively:

1 $$\begin{aligned} \begin{aligned} {\left\{ \begin{array}{ll} &{}S_c \propto {1 \over k_c} \sum _{i}{M_{ci}} {\propto {Y_i} \over k'_i}\\ &{}Y_i \propto {1 \over k'_i} \sum _{c}{M_{ci}} {S_c \over k_c} \end{array}\right. } \end{aligned} \end{aligned}$$

where $k_c={\sum _{i}{M_{ci}}}$ is the degree of the countries and $k'_i=\sum _{c}{M_{ci}}/K_c$ is the level of equality indicator *i* accounting for the relative performance of countries in this equality goal. Note that computing the $\text {EU MECI} \equiv S_c$ requires the calculation of the $Y_i$ values and the indicator-specific weights, $w_i=Y_i/k'_i$. The $Y_i$ (and $w_i$) values are functional to the computation of the EU MECI and vice versa. The data-driven weight $w_i$ can be interpreted as a measure of difficulty of improving equality indicator *i* at the EU level.

Table 1 lists the indicators with the five highest and lowest weighting scores (the complete list is available upon request). The *GENEPY* approach suggests that the equality indicator ‘*P90/P10 ratio of PISA scores in mathematics*’ (code 5) in the ‘*Knowledge and skills*’ dimension is the measure with the biggest limitations in terms of improvement when chasing a more equitable Union. On the other end of the spectrum is ‘*Relative gap in average hours of full-time employment worked per week (female over male)*’ (code 151) in the ‘*Working life*’ dimension, which reflects a (generally) good performance by all Member States.

These findings are of high importance in guiding policy making both at the EU and the national level. More effort through policy interventions is needed in improving the EU’s performance in PISA scores and this effort should be initiated and focused on the Member States with low EU MECI scores (see next Section).

**Table 1:** Indicators with the top and bottom five weights

Rank	Code	Dimension	Indicator	Weight ($w_i$)
1	5	Knowledge and skills	P90/P10 ratio of PISA scores in mathematics	0.1801
2	209	Cultural life and recreation	Odds ratio of going to the cinema (elderly over middle-aged adult, adjusted)	0.1535
3	166	Working life	Odds ratio of being a low-wage earner (tertiary over less than upper secondary education)	0.1523
4	37	Health	Odds ratio of self-reported high health status (elderly over middle-aged adult, adjusted)	0.1420
5	187	Cultural life and recreation	Absolute Gini index of satisfaction with leisure time	0.1229
173	16	Knowledge and skills	Odds ratio of NEET rates (female over male)	0.0182
174	28	Health	Legal framework not protecting women’s reproductive health and rights	0.0178
175	105	Material living conditions	Odds ratio of household not being able to afford an unexpected expense (elderly over middle-aged adult, adjusted)	0.0175
176	199	Cultural life and recreation	Odds ratio of practising artistic activities as a hobby (young adult over middle-aged adult, adjusted)	0.0174
177	151	Working life	Relative gap in average hours of full-time employment worked per week (female over male)	0.0170

## A Snapshot of EU Member States Responses to the President’s Call

**Fig. 3:**
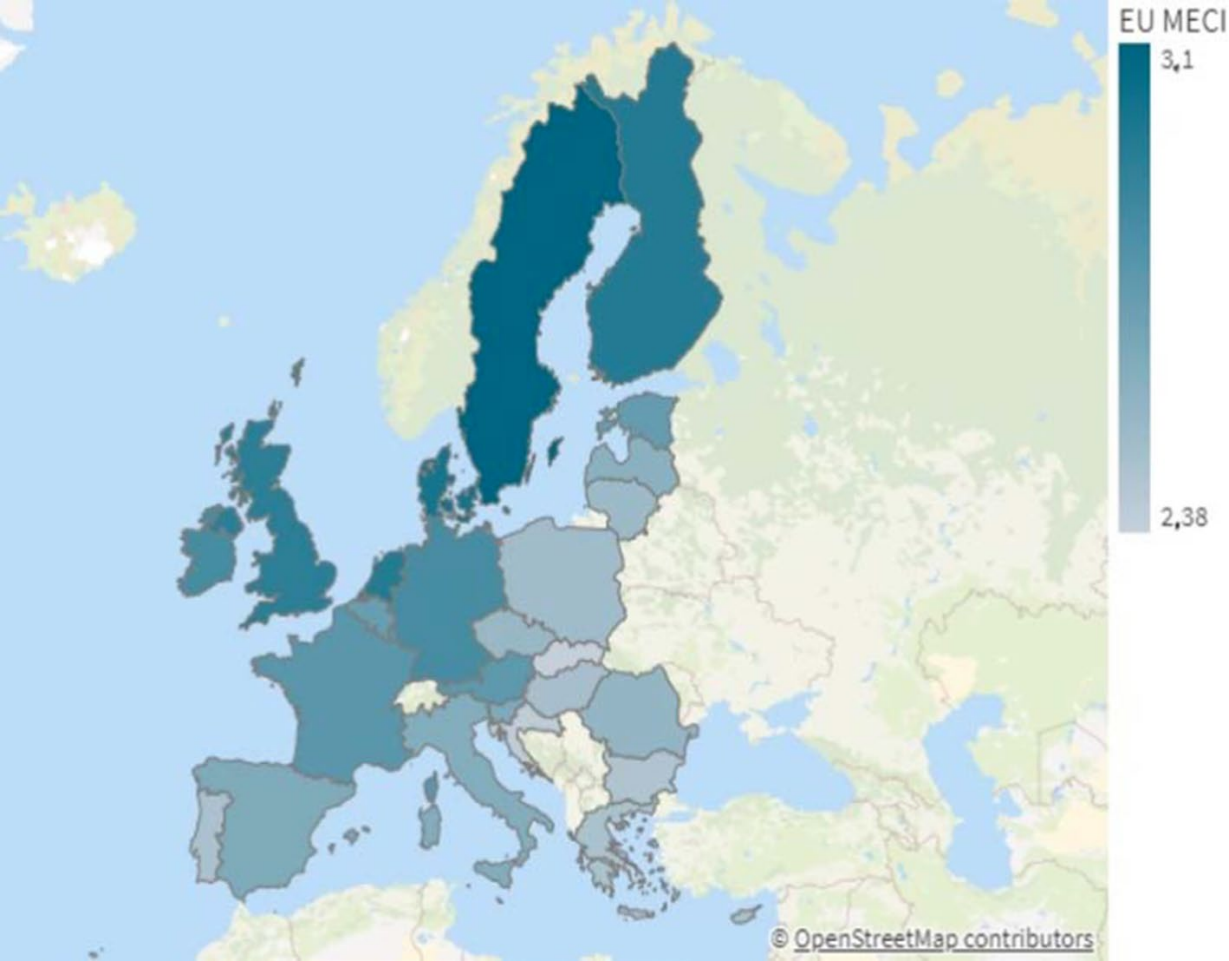
EU MECI across Member States. Countries depicted in dark blue have a high EU MECI value, indicating more equitable societies

Figure 3 maps the EU MECI across the Member States, providing insight about where countries stand in terms of building a Union of equality. Table 3 in Appendix A. shows the ranking and values.

**Fig. 4:**
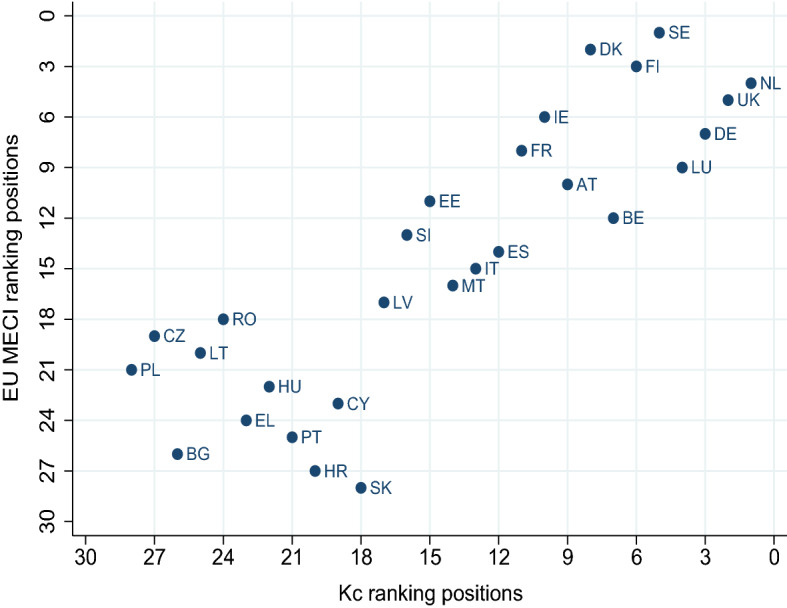
EU MECI versus countries’ degrees, $k_c$. Countries lying along the diagonal share the same EU MECI rankings, with an index based on equal weights for countries and indicators

**Fig. 5:**
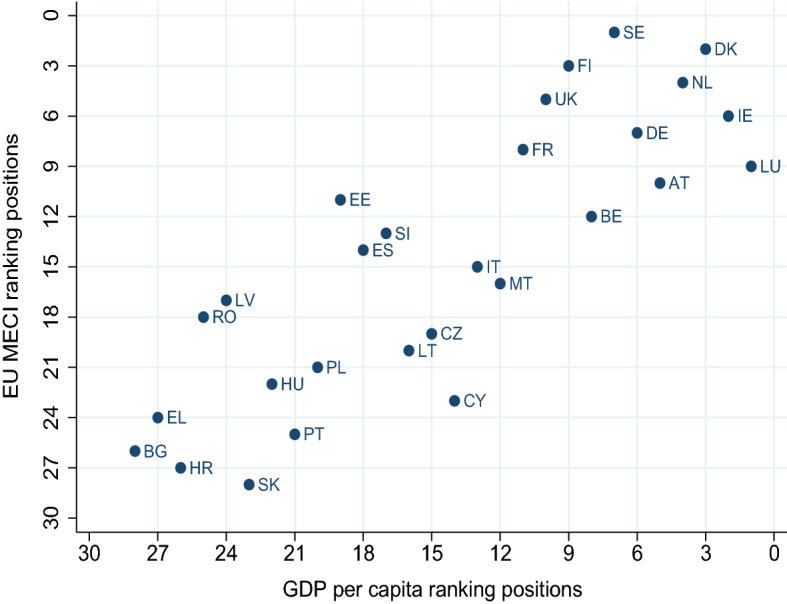
EU MECI versus GDP per capita. GDP per capita in PPP constant 2017 international $ values; Year: 2020; Data compiled from multiple sources by World Bank; published online at https://ourworldindata.org/ Roser (2013)

**Fig. 6:**
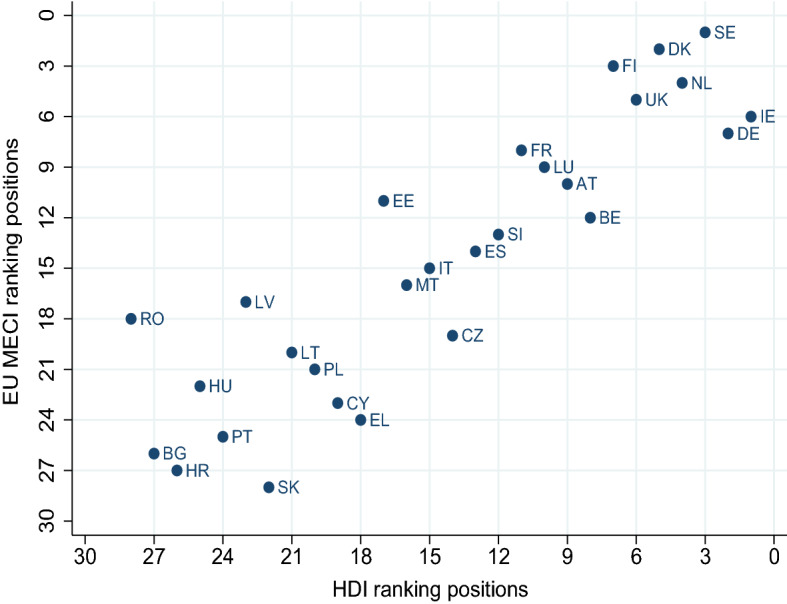
EU MECI versus HDI. Human Development Index (HDI); Year: 2017; Data from United Nations Development Programme, Human Development Report 2020; published online at https://ourworldindata.org/ Roser (2014)

**Fig. 7:**
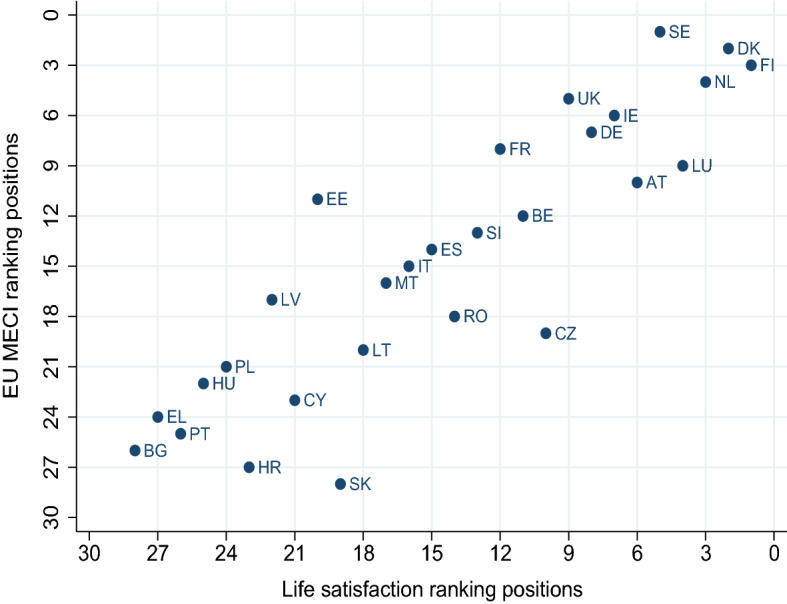
EU MECI versus Life satisfaction. Self reported life satisfaction; Year: 2020; Data from World Happiness Report 2022; published online at https://ourworldindata.org/ Ortiz-Ospina and Roser (2013)

**Table 2:** ‘Good performers’ versus ‘poor performers’ in indicators with the top and bottom three weights

Indicator code	5	209	166	105	199	151
($w_i$)	(0.1801)	(0.1535)	(0.1523)	(0.0175)	(0.0174)	(0.0170)
*High EU MECI*						
Sweden	10.61	25.18	20.62	90.97	79.66	93.19
Denmark	15.31	26.34	4.08	88.73	93.25	89.67
Finland	15.15	12.28	10.54	88.52	96.00	90.95
Netherlands	10.49	11.12	35.31	91.37	93.40	74.58
*Low EU MECI*						
Portugal	6.08	7.87	0.68	92.65	90.90	93.82
Bulgaria	0.00	0.00	6.25	0.00	97.25	100.0
Croatia	9.70	4.41	0.36	96.91	86.87	97.97
Slovakia	4.42	1.47	1.41	99.25	87.40	96.06

For a description of the indicators, see reference ((Alberti et al., 2021), Annex)

Sweden appears at the top of the ranking of the most equal countries, followed by Denmark, Finland and the Netherlands. These top four Member States show relatively good performance on the indicators with the top three weights (5, 209 and 166) compared to the low EU MECI countries: Portugal, Bulgaria, Croatia and Slovakia (Table 2). This might imply that the relatively ‘good performers’ in equality goals that are challenging have invested more effort towards the improvement and achievement of equitable development compared to the other Member States. On the other hand, the relatively ‘poor performers’ tend to do well only on the indicators with the lowest limitations of improvement at the EU level, such as the indicators with codes 105, 199 and 151.

Figure 4 plots the EU MECI ranking positions of countries and their degree rankings. This figure reveals the differences in the rankings when instead of using more classical approaches based on the countries’ degrees, $k_c$ (i.e., the sum of all indicators’ performances assuming equal weights), one follows the complex network approach proposed here. The figure visually reveals the two-tier Europe, with “low” and “high” performers. The *“geography curse”* of the east and the south, differentiates the group located in the bottom left corner of the figure, from the rest of the EU. At the same time, the traditionally high achievers of the strong core of the Union remain at the top of the rankings. The geographical segregation or the EU’s internal East-West divide is a well established finding in various areas of life in the EU (Ulceluse & Bender, 2022). In fact, a strand of this literature warns against the implicit hierarchical structure of the EU predating its formation (Ulceluse & Bender, 2022; Antonucci & Varriale, 2020), built on strong trade-offs benefiting the core countries at the expense of the periphery (Underdeveloped Europe, 1979). The verification of such geographical segregation through the EU MECI should raise a more boisterous alarm to those in charge of public policies in the Union.

To shed some further light on our findings, in Figs. 5, 6 and 7 we compare the computed EU MECI rankings vs the *GDP per capita* and well being indicators not included in the MIMF, such as the *HDI* and *Life Satisfaction* respectively. The astonishingly high similarity level in all figures lies in the replication of the segregation of the two tiers of countries on one hand, and the clearly positive correlation between equality performance, *GDP per capita*, *HDI* and *Life Satisfaction*.

The differences between the EU MECI and *GDP per capita* exhibited in Fig. 5, reflect that, despite the high correlation between their rankings, there is valuable information captured by the novel and comprehensive *GENEPY* approach, able to partially shake the traditional and deep-rooted per capita GDP country ranking. This finding might imply the need for adjustments in the prioritization of areas and/or countries towards which EC policies are directed.

Furthermore, a closer observation of the dispersion of countries around an imaginary 45 degree line reveals that countries lying on the right of the line exhibit a higher ranking in terms of *GDP per capita* versus the EU MECI, stressing the significant limitation of a single indicator in informing country performance when it comes to equality. Figures 6 and 7 verify the geographical segregation and the positive correlation discussed above, however exhibit less dispersion, verifying that both indicators, namely the *HDI* and *Life Satisfaction*, capture information more comparable to the EU MECI. All in all, we conclude that the EU MECI performs at least as well as the standard socio-economic indicators used in the literature, while being able to encompass rich information hidden in its structure, offering new insights.

## Conclusions

The study of a complex system, such as the multifaceted phenomenon of equality at the EU level, which percolates through various dimensions of citizens’ well-being from material living conditions to health, education and culture, requires a complex systems approach. In this work, based on the data measured by the EU MIMF, we devise the EU MECI, derived by structuring the EU MIMF data as a bipartite network of countries and indicators. The EU MECI introduces an innovative way to measure and address inequalities by applying the economic complexity methodology on the countries-indicators network’s centrality metrics and exploring (a) the interactions across the equality indicators and (b) the interplay between Member States and equality measures, while the corresponding weights of countries and indicators are determined endogenously by the data.

Our ranking of indicators, based on the data-driven weights, reveals the equality goals, and implicitly the key life dimensions, that policy makers should concentrate their efforts on in order to devise action plans towards a more equitable European society. What is more, the EU MECI, offers a comprehensive tool for monitoring and analysing multidimensional inequalities in the EU, that allows for a geographical prioritization of the corresponding policy interventions, where EU and Member State strategies should align and collaborate. Our aggregate ranking captured by the EU MECI verifies the geographical two-tier segregation of EU countries, while highlights the advantages, in terms of actionable insights, that the network science offers as compared to traditional approaches with neutral weights.

From a policy perspective, the proposed analysis complements the EU MIMF by introducing an additional arrow into the policymaker’s quiver of effective policies to tackle unfair social inequalities. It does so by identifying—through data-driven techniques—the areas in which Member States find it more difficult to reduce existing inequalities.

Building a Union of equality is a core topic in the von der Leyen Commission’s policy debate, and the analysis presented in this work can play an important role in the formulation of evidence-based policy responses to rising social disparities.

## Disclaimer

The information and views set out in this paper are those of the authors and do not necessarily state or reflect the official opinion of the European Commission. The scientific output expressed does not imply a policy position of the European Commission. Neither the European Union institutions and bodies nor any person acting on their behalf may be held responsible for the use, which may be made of the information contained therein.

## Figures and Tables

**Fig. 8:**
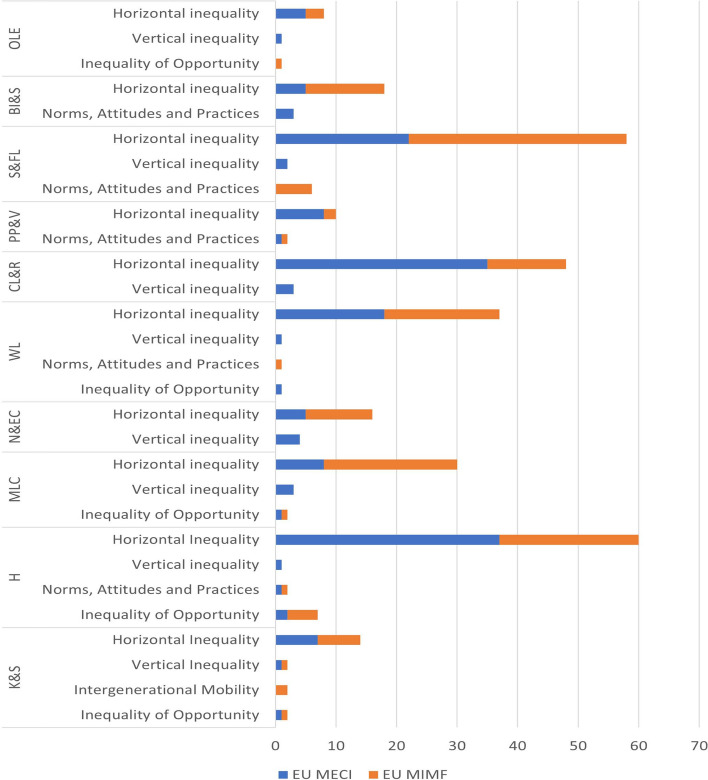
The EU MIMF versus EU MECI set of indicators. The number of indicators used in the development of EU MECI versus the number of indicators included in the EU MIMF per measuring approach and life dimension; $K \& S$: ‘*Knowledge and skills*’; $N \& EC$: ‘*Natural and environmental conditions*’; $S \& FL$: ‘*Social and family life*’; $BI \& S$: ‘*Bodily integrity and safety*’; *MLC*: ‘*Material living conditions*’; *WL*: ‘*Working life*’; $CL \& R$: ‘*Cultural life and recreation*’; *H*: ‘*Health*’; $PP \& V$: ‘*Political participation and voice*’; *OLE*: ‘*Overall life experience*’

## Data Availability

The data that support the findings of this study are available in the European Commission’s *EU Multidimensional Inequality Monitoring Framework*,[https://composite-indicators.jrc.ec.europa.eu/multidimensional-inequality/documentation]

## Appendix A

See Fig. 8.

See Tables 3 and 4.

**Table 3:** The EU MECI of Member States (ranking and values)

Country	EU MECI rank	EU MECI value
Sweden	1	3.0985
Denmark	2	2.9968
Finland	3	2.9513
Netherlands	4	2.9483
United Kingdom	5	2.9264
Ireland	6	2.8326
Germany	7	2.8305
France	8	2.7692
Luxembourg	9	2.7583
Austria	10	2.7496
Estonia	11	2.7322
Belgium	12	2.7175
Slovenia	13	2.6394
Spain	14	2.6380
Italy	15	2.6202
Malta	16	2.6157
Latvia	17	2.5931
Romania	18	2.5605
Czech Republic	19	2.5425
Lithuania	20	2.5253
Poland	21	2.5139
Hungary	22	2.4906
Cyprus	23	2.4793
Greece	24	2.4777
Portugal	25	2.4770
Bulgaria	26	2.4600
Croatia	27	2.4063
Slovakia	28	2.3820

**Table 4:** The final list of indicator codes included in the analysis

1	59	105	171	224	278
5	60	107	186	225	279
7	61	126	187	227	281
11	62	127	188	228	284
12	63	128	189	230	285
13	64	129	190	231	286
16	65	130	191	232	287
19	67	131	192	233	289
20	69	132	193	235	292
21	72	133	195	236	293
23	75	135	198	239	294
28	76	146	199	240	295
30	77	147	200	241	297
31	78	149	201	242	316
33	80	151	203	243	317
34	83	152	204	244	318
35	84	153	206	245	321
36	85	154	207	247	322
37	86	155	208	248	323
39	88	156	209	256	324
42	91	157	211	257	326
43	93	158	212	258	338
44	94	159	214	259	339
45	95	161	215	260	340
47	96	163	216	261	341
50	97	165	217	262	342
51	98	166	219	263	344
52	100	167	220	265	
53	103	168	222	276	
55	104	169	223	277	

For indicator details, see reference ((Alberti et al., 2021), Annex)

## Footnotes

1 The United Kingdom ceased to be an EU Member State on 31 January 2020, but given that the measurement/source year of the EU MIMF indicators is before 2021, we use the term ‘Member State’ to refer also to the UK

2 https://ec.europa.eu/info/strategy/priorities-2019-2024/economy-works-people_en.

3 https://composite-indicators.jrc.ec.europa.eu/multidimensional-inequality.

4 The indicator missing is the gender pay gap (code 150), which as also shown in Figure 3.2 of the EU MIMF report (Alberti et al., 2021) is not available for Italy.

## References

[CR1] Acemoglu D, Johnson S, Robinson JA (2005). Institutions as a fundamental cause of long-run growth. Handbook of Economic Growth.

[CR2] Alberti, V., Banys, K., Caperna, G., De Sorbo, M., Fregoni, M., Havari, E., Kovacic, M., Lapatinas, A., Litina, A., Valentina, M., Moura, C., Neher, F., Panella, F., Peragine, V., Pisoni, E., Stuhler, J., Symeonidis, K., Verzillo, S., Boldrini. M. (2021). In M. Domínguez-Torreiro, E. Papadimitriou (Eds.), *Monitoring multidimensional inequalities in the European Union. *Luxembourg: Publications Office of the European Union.

[CR3] Allesina S, Tang S (2012). Stability criteria for complex ecosystems. Nature.

[CR4] Antonucci L, Varriale S (2020). Unequal europe, unequal brexit: How intra-european inequalities shape the unfolding and framing of brexit. Current Sociology.

[CR5] Atkinson AB, Brandolini A (2010). On analyzing the world distribution of income. The World Bank Economic Review.

[CR6] Bascompte J, Jordano P, Melián CJ, Olesen JM (2003). The nested assembly of plant-animal mutualistic networks. Proceedings of the National Academy of Sciences.

[CR7] Le Blanc D (2015). Towards integration at last? The sustainable development goals as a network of targets. Sustainable Development.

[CR8] Bonacich P (1987). Power and centrality: A family of measures. American Journal of Sociology.

[CR9] Copeland, A.H. (1951). A reasonable social welfare function, Tech Rep, mimeo, 1951. University of Michigan.

[CR10] Ferreira, F.H., Peragine, V. (2016). Individual responsibility and equality of opportunity, The Oxford handbook of well-being and public policy.

[CR11] Hidalgo CA (2021). Economic complexity theory and applications. Nature Reviews Physics.

[CR12] Hidalgo CA, Hausmann R (2009). The building blocks of economic complexity. Proceedings of the National Academy of Sciences.

[CR13] Ladyman J, Lambert J, Wiesner K (2013). What is a complex system?, European Journal for. Philosophy of Science.

[CR14] Niño-Zarazúa M, Roope L, Tarp F (2017). Global inequality: Relatively lower, absolutely higher. Review of Income and Wealth.

[CR15] Nussbaum, M.C. (2001). Women and human development: The capabilities approach, Vol. 3, Cambridge University Press.

[CR16] OECD, Howapos;s Life? 2017, (2017).10.1787/how_life-2017-en. https://www.oecd-ilibrary.org/content/publication/how_life-2017-en

[CR17] OECD, For Good Measure, (2018) . 10.1787/9789264307278-en. https://www.oecd-ilibrary.org/content/publication/9789264307278-en

[CR18] Ortiz-Ospina, E., Roser, M. (2013). Happiness and life satisfaction, Our World in DataHttps://ourworldindata.org/happiness-and-life-satisfaction.

[CR19] Ravallion, M., Thorbecke, E., & Pritchett, L. (2004). Competing concepts of inequality in the globalization debate [with comments and discussion], In: Brookings trade forum, JSTOR, 2004, pp. 1–38.

[CR20] Roser, M. (2013). Economic growth, Our World in DataHttps://ourworldindata.org/economic-growth.

[CR21] Roser, M. (2014) Human development index (hdi), Our World in DataHttps://ourworldindata.org/human-development-index.

[CR22] Sbardella A, Pugliese E, Pietronero L (2017). Economic development and wage inequality: A complex system analysis. PloS one.

[CR23] Sciarra C, Chiarotti G, Ridolfi L, Laio F (2020). Reconciling contrasting views on economic complexity. Nature Communications.

[CR24] Sciarra C, Chiarotti G, Ridolfi L, Laio F (2021). A network approach to rank countries chasing sustainable development. Scientific Reports.

[CR25] Sen, A (1999) The ends and means of development, Development as freedom 35–53.

[CR26] Sen, A (1985). Commodities and capabilities (professor dr. p. hennipman lectures in economics; amsterdam; new york, north-holland; elsevier science pub. co, sole distributors for the usa and canada.).

[CR27] Tabellini G (2008). Institutions and culture. Journal of the European Economic association.

[CR28] Tacchella A, Cristelli M, Caldarelli G, Gabrielli A, Pietronero L (2012). A new metrics for countries’ fitness and products’ complexity. Scientific reports.

[CR29] (UN), U.N. (2020) Shared Responsibility, Global Solidarity: responding to the socio-economic impacts of Covid-19, UN.

[CR30] Ulceluse M, Bender F (2022). Two-tier eu citizenship: Disposable eastern european workers during the covid-19 pandemic. Organization.

[CR31] Underdeveloped Europe : studies in core-periphery relations (1979). Harvester studies in development ; no.1, Harvester Press, Hassocks.

[CR32] Williamson OE (2000). The new institutional economics: taking stock, looking ahead. Journal of economic literature.

